# Investigating the Safety and Efficacy of Platelet-Rich Plasma (PRP) Treatment for Female Androgenetic Alopecia: Review of the Literature

**DOI:** 10.3390/medicina57040311

**Published:** 2021-03-25

**Authors:** Santo Raffaele Mercuri, Giovanni Paolino, Matteo Riccardo Di Nicola, Laura Vollono

**Affiliations:** 1 Unit of Dermatology, IRCCS San Raffaele Hospital, 20132 Milan, Italy; mercuri.santoraffaele@hsr.it (S.R.M.); paolino.giovanni@hsr.it (G.P.); 2 Via Po 102, 00198 Rome, Italy; laura.vollono@gmail.com

**Keywords:** platelet-rich plasma, alopecia, hair loss, androgens, PRP, hair follicles

## Abstract

*Background*: female androgenetic alopecia (FAGA) is a common cause of non-scarring alopecia in women, affecting approximately 40% of women by age 50, bearing a significant psychosocial burden on affected patients. Platelet-rich plasma (PRP) has been widely investigated as a potential effective treatment for several dermatological conditions, including male androgenetic alopecia (MAGA). However, few studies have been conducted focusing on the use of PRP in FAGA. The aim of this review was to identify reports that investigated the use of PRP for the treatment of FAGA. *Methods*: Electronic databases of MEDLINE, EMBASE, and Cochrane Central Register of Controlled Trials (CENTRAL) from inception to September 2020 have been searched using different combinations of the following terms: “androgenetic alopecia,” “FAGA,” “female pattern hair loss,” “platelet-rich fibrin,” “platelet-rich plasma,” and “PRP”. *Results and conclusions*: Eight (*n* = 8) clinical studies consistent with our research were identified. A total of 197 subjects has been enrolled in the included studies. All of them were adult female patients (mean age: 38.9) affected by female pattern hair loss. PRP is a well-tolerated procedure which showed promising results in males-only and mixed populations of AGA patients. PRP showed to produce high levels of satisfaction and improvement in the quality of life in patients affected by FAGA. In the light of this evidence, PRP may be proposed in patients who did not respond or did not tolerate topical minoxidil, as well as in combination with topical and oral treatments.

## 1. Introduction

Androgenetic alopecia (AGA) is an encompassing term for non-scarring hair loss that typically begins in adult life, mediated by the action of androgens on genetically susceptible hair follicles [1]. Although the histopathological findings are identical in both sexes, clinical presentation and therapeutic implications differ considerably, hence the term female androgenetic alopecia (FAGA) advocated by many authors [2,3,4].

### 1.1. FAGA: Clinical Features, Pathogenesis and Current Therapeutic Options

FAGA is a common cause of non-scarring alopecia in women, affecting approximately 40% of women by age 50 [5,6]. FAGA is a complex polygenic trait, with a high prevalence and wide range of expressed phenotypes. However, fewer genetic association studies are available for FAGA compared with male AGA. Polymorphisms in the androgen receptor gene on the X chromosome, as well as in the genes encoding for the cytochrome p450 alpha-aromatase enzyme, 3β-hydroxysteroid dehydrogenase, and vitamin D receptor have been associated with the development and persistence of FAGA, determining the age of onset, progression, patterning and severity [7,8,9,10,11].

Androgens play a pivotal role in the development of FAGA. The dermal papilla of the pilosebaceous unit is able to synthesize and metabolize a wide range of androgens, with subsequent alteration of soluble factors regulating the growth and activity of hair follicles keratinocytes [12,13].

In genetically predisposed individuals, hair follicles are oversensitive to dihydrotestosterone (DHT), which is converted from testosterone by the 5α-reductase type II enzyme. DHT binds androgens receptors with an approximate fivefold greater affinity than its precursor, activating different signaling pathways resulting in increased secretion of transforming growth factor-B from keratinocytes, apoptosis of the dermal papilla (DP) cells, and inability to stimulate proliferation of keratinocytes [14,15,16].

These processes compromise the ability of DP cells to undergo hair follicle cycle remodeling and new hair shaft growth, resulting in hair follicle miniaturization.

In affected individuals, the hair cycle is altered with a progressively decrease in the anagen phase with each successive cycle, while the telogen phase remains constant. Concurrently, the size of the hair follicles decreases. This results in progressive shorter and thinner terminal hair, replaced by short fine vellus hair. The number of follicles per unit area remains the same.

Clinically, FAGA is characterized by diffuse thinning of the crown and vertex region, with the front hairline variably affected. Mild recession of the frontal hairline is rare in pre-menopausal women, being observed in 37% of post-menopausal age [17]. The occipital area is spared, being the significant difference among hair density on the crown and in the occipital area a clue for diagnosis. This clinical pattern is due to the different characteristics of hair follicles in the different areas of the scalp. One of the main differential diagnoses of FAGA is female pattern hair loss (FPHL), which is a term used to describe the decrease in central scalp hair density that occurs in a number of females after puberty. Although there is not a clear difference between FAGA and FPHL, some authors call female alopecia with androgen increase female FAGA and call female alopecia without androgen increase FPHL [18,19,20]. In this review, we used the general term FAGA to omologate the search strategy.

Compared to men, women affected by androgenetic alopecia experience overall less severe hair loss. This is due to the fact that expression of 5α-reductase and androgen receptors in female hair follicles are approximately half the levels of the male counterpart. Additionally, female hair follicles express higher levels of aromatase compared to male hair follicles, resulting in increased local production of estradiol from testosterone and subsequent less formation of DHT [4,21].

Although Ludwig assumed that FAGA and MAGA represented the same androgen-dependant condition with different clinical presentations between sexes, this has been widely questioned later on [22]. Many authors have argued that in spite of identical histopathology, FAGA and MAGA are two separate entities characterized by different peak ages of onset, rates of prevalence, and patterning of hair loss.

Women with hyperandrogenism may present with patterned hair loss and hirsutism, however, most women with FAGA have normal levels of androgens [23,24].

Likewise, it has been observed that women without circulating androgens may also develop FAGA, further questioning the role of androgen levels in the development of FAGA [25]. This could explain the variable response of FAGA to androgen inhibition therapy [12].

For all these reasons, we believe that clinical studies investigating the effectiveness of potential treatments for FAGA should be focused on populations composed only of female subjects. In our opinion, this would allow us to obtain more significant results compared to those conducted on mixed populations. In this paper, we reviewed only clinical studies that were conducted on populations composed strictly by female subjects.

Although a benign condition, hair loss in FAGA may have a significant psychosocial impact, resulting in reduced health-related quality of life (HrQoL) and anxiety and depression in women with FAGA [26,27].

Clinical assessment and thoughtful evaluation of FAGA are important for several reasons. First, careful clinical and eventual histopathological evaluation are crucial in order to exclude other conditions with a clinical appearance similar to that of FAGA, such as telogen effluvium, diffuse and incognita alopecia areata, fibrosing alopecia in pattern distribution, and cicatricial pattern hair loss.

Second, assessing the stage of the disease is necessary in order to choose the most appropriate therapeutic management. Furthermore, as FAGA is a slowly progressive disease, assessment according to objectives criteria is needed in order to monitor therapeutic response and reassure the patient, who often tends to get used to the improved appearance of her hair over months or years and underestimate the results achieved.

Ludwig first introduced clinical grading of FAGA, describing three stages of severity ranging from rarefaction of the hair on the crown (I) to near-total baldness (III) [22].

However, a clinical diagnosis based only on the Ludwig classification may underestimate early FAGA and overestimate advanced FAGA.

Later on, a 5-point visual analog grading scale based on photographs obtained by a stereotactic device mounted at fixed distance from the scalp was found to be more precise in assessing the progression of the disease and the treatment response, with the advantage of a high reproducibility [28].

Dermoscopy and videodermoscopy of the hair and scalp (trichoscopy) were introduced in the early 2000s [29,30,31]. In FAGA, hair shaft thickness heterogeneity (anisotrichia) in patterned areas of the scalp is the most prominent trichoscopic feature. Trichoscopy has been proved to be a useful tool in the management of FAGA, especially in monitoring therapeutic response by comparison of seriate images [32].

The above-listed tools are widely used in routine clinical practice. However, objective methods allowing to perform quantitative evaluation of hair growth are required for clinical studies. The global photographic assessment is a semi-objective tool commonly used in clinical studies, evaluated by experts blinded to treatment and time [33].

Phototrichogram and contrast-enhanced phototrichogram are non-invasive, highly reproducible photographic systems capable of measuring the parameters related to hair growth and loss of hair such as number of hairs, hair density, hair diameter, percentage of hair in anagen and telogen phase, and percentage of terminal and vellus hair [34]. These techniques have been proved to be equally sensitive as scalp biopsies for hair detection and growth staging, being widely used in clinical studies [35,36]. The introduction of TrichoScan^®^, a more modern fully-automated technique combining epiluminescence microscopy and automated digital image analysis further improved intra- and inter-evaluator variability and reliability, making an excellent candidate for clinical trials [37,38,39,40,41].

FAGA is a slowly progressive disease, with a progression rate estimated to be around 10% per year [41]. However, a significant percentage of hair density must be lost before the condition becomes clinically evident, resulting in delay of diagnosis and treatment. Several treatments are effective in arresting the progression of hair thinning and, in some cases, stimulate partial regrowth, however, complete regrowth of hair does not usually occur.

Various treatment options have been attempted and are being studied for FAGA.

To date, the only agent approved by the US Food and Drug Administration (FDA) is topical minoxidil [3,42]. Other therapeutic options currently available include systemic drugs such as oral minoxidil, finasteride, dutasteride, spironolactone, flutamide, cyproterone acetate [33]. All treatments must be prolonged for long periods in order to achieve and maintain results. Progression of the disease is expected in case of withdrawal of treatment. Although generally well-tolerated, all these treatments can be time-consuming in daily routine and bear side effects, leading many patients to seek alternative physical treatments such as low-level laser (light) therapy, microneedling, and platelet-rich plasma (PRP) injection [33,43]

Because of the growing interest among both patients and the medical community, we conducted a review in order to investigate the potential usefulness and recommended protocols of PRP injections for the treatment of FAGA.

### 1.2. Platelet Rich Plasma (PRP)

Platelet-rich plasma is a plasma fraction characterized by a 3- to 7-fold increased concentration of platelets compared to whole blood [44]. It is obtained by collecting approximately 10–60 mL of peripheric blood the same day of treatment. Anticoagulants are usually added to prevent coagulation and premature secretion of alpha granules. After centrifugation, different coats characterized by different composition in blood cells can be identified in the tube. Depending on the coats harvested, four different types of preparation can be obtained: (I) Pure Platelet Rich Plasma, a leukocyte-poor PRP with low-density fibrin network after activation; (II) Leucocyte-PRP, rich in leukocytes and with low fibrin network; (III) Pure Platelet-Rich Fibrin, poor in cells and dense in fibrin; (IV) Leucocyte and Platelet-Rich Fibrin, rich in cells and with high-density fibrin network [45,46].

**Figure 1:**
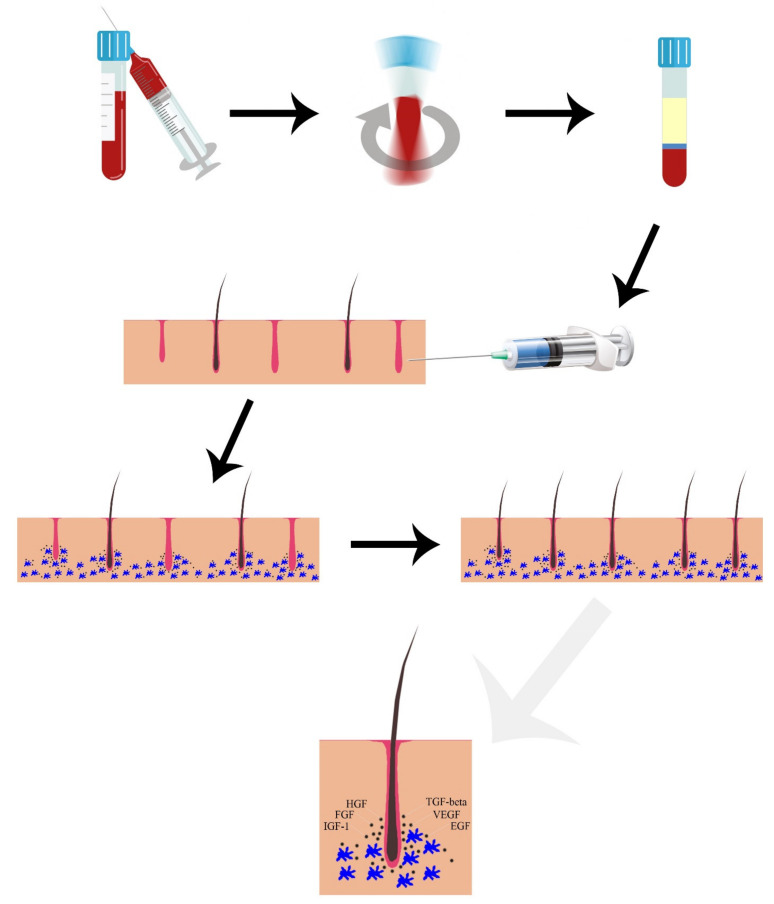
Mechanism of action of PRP in FAGA. The release of VEGF, PDGF, EGF, TGF-β, and FGF promotes angiogenesis, providing hair follicles with nutrients and oxygen. VEGF = vascular endothelial growth factor; PDGF = platelet-derived growth factor; EGF = epidermal growth factor; TGF-β = transforming growth factor beta; FGF = fibroblast growth factor; HGF = hair growth factor; IGF-1 = Insulin-like growth factor 1. Vector files downloaded from Vecteezy.com.

Through the secretion of growth factors, adhesion molecules, and chemokines contained in platelet’s alpha granules, PRP is able to promote cell differentiation, proliferation, and regeneration. Platelet-derived growth factor (PDGF) type a and b, transforming growth factor (TGF) type α and β, vascular endothelial growth factor (VEGF), epidermal growth factor (EGF), fibroblast growth factor (FGF), connective tissue growth factor (CTGF) and insulin-like growth factor-1 (IGF-1) are actively secreted by platelets after 10 min from their activation. Furthermore, platelets are able to release numerous anti-inflammatory cytokines, such as interleukin-1 receptor antagonist (IL-1ra), soluble tumor necrosis factor (TNF) receptor (sTNF-R) I, interleukin (IL)-4, IL-10, IL-13 and interferon γ [47] (Figure 1). Host dermal collagen and endogenous thrombin are able to activate PRP (“non-activated PRP” or NA-PRP) however, calcium gluconate, calcium chloride, or exogenous thrombin can be added before administration (“activated PRP” or AA-PRP).

As impaired cell signaling, altered growth factor and cytokine production contribute significantly to AGA, there is great interest in the use of PRP for FAGA treatment [48]. The release of these growth factors leads to activation of the extracellular signal-regulated kinase (ERK) and protein kinase B (Akt) signaling pathways, resulting in accumulation of β-catenin and FGF-7 [49]. These factors are able to promote proliferation of DP cells in the bulge area, resulting in induction of follicular stem cell differentiation and stimulation of the transition from telogen to anagen phase in hair follicles. The activation of anti-apoptotic signaling pathways, such as those of Bcl-2 and Akt, prevent catagen phase with subsequent lengthening of the anagen phase, allowing the hair to grow [50]. Furthermore, the release of VEGF, PDGF, EGF, TGF-β, and FGF promotes angiogenesis, providing hair follicles with nutrients and oxygen [51]. The potential efficacy of platelet concentrates in promoting wound healing and tissue regeneration has been at the center of scientific debate over the past few decades [52].

## 2. Materials and Methods

**Figure 2:**
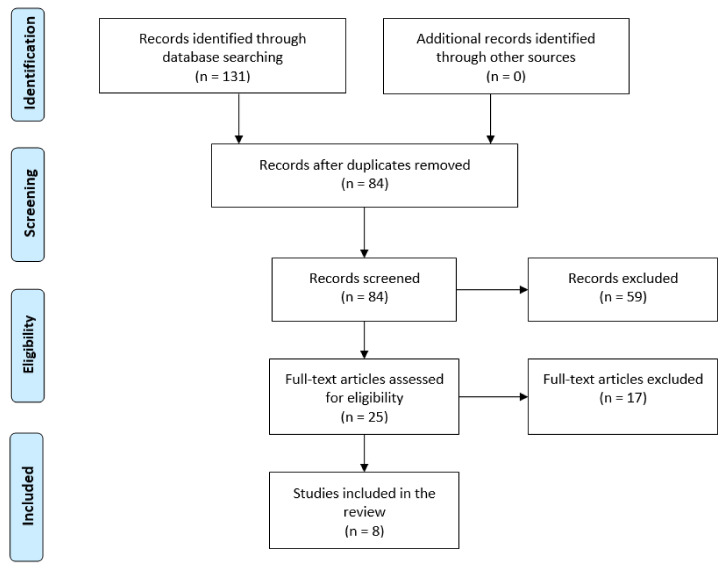
Flow chart of search strategy.

We searched the literature to identify studies that investigated the use of PRP for the treatment of FAGA. The electronic databases of PubMed, MEDLINE, EMBASE, and Cochrane Central Register of Controlled Trials (CENTRAL) from inception to September 2020 have been searched using different combinations of the following terms: “androgenetic alopecia,” “FAGA,” “female pattern hair loss,” “platelet-rich fibrin,” “platelet-rich plasma,” and “PRP” (Figure 2). For this review, we followed the Preferred Reporting Items for Systematic Reviews and Meta-Analyses (PRISMA) guidelines, where feasible [53]. As many authors consider FAGA a separate entity from male androgenetic alopecia, studies conducted on a mixed population including both sexes were excluded in order to allow correct evaluation of the results.

## 3. Results

Eight (*n* = 8) clinical studies consistent with our research were identified. Among them, only one (*n* = 1) was an ongoing clinical trial, with not yet published results. A total of 197 subjects has been enrolled in the included published studies. All of them were adult females (mean age: 38.9) who were diagnosed with female pattern hair loss. Study design, number of enrolled patients, Ludwig score, type of intervention, eventual concurrent other treatments, PRP preparation process and technique, number and times of sessions, duration of the study, assessment criteria, endpoints, results, time of follow-up and reported side effects of all included studies are summarized in Table 1.

Enrolled patients presented with a Ludwig stage ranging from I to III, corresponding to appreciable thinning of the crown hair (I), increased territorial involvement and scalp visibility (II), and total denudation of the areas involved in I and II (III) [54]. In all studies, patients were either naive or underwent a wash-out period from any treatment ranging from 2 weeks to 1 year. A number of sessions performed ranged from 1 to 6.

Time session varied considerably among studies, ranging from every 2 weeks to every 3 months. The mean duration of the studies (published data) was 30 weeks, ranging from 14 weeks to 1 year. As to clinical effectiveness, endpoints of the studies were changed in global clinical appearance, hair count, hair density (number of hairs/cm^2^), hair diameter, total thickness, hair mass index, vellus hair count, vellus hair density (number of vellus hairs/cm^2^). Assessment methods used were global photography, hair pull test, Cohen hair check system, magnified photography, phototrichograms (variably associated with Trichoscan^®^ and Follioscope^®^ software).

As to the patient’s perspective, patients’ quality of life and satisfaction were investigated through a 16-item quality-of life (QOL) questionnaire, 7-item questionnaire, patient’s satisfaction scale, and custom surveys.

After PRP treatment, hair count was found to be significantly increased in 4 studies [55,56,57,58]. An increase was found greater when compared to saline injections in 2 studies administering NA-L-PRP and AA-L-PRP [50,52]. Conversely, one further study reported no difference in hair count after NA-P-PRP compared to placebo [59]. Combination treatment of PRP+ polydeoxirybonucleotide (PRDR) injections resulted in a greater increase in hair count compared to PRDN injections only. On the other hand, improvement in hair count after u-PRP was found to be milder than the one achieved after treatment with topical minoxidil [60].

As to hair diameter, it was found significantly increased in 4 studies [55,56,57,58]. In particular, improvement in hair diameter achieved after combination treatment of NA-L-PRP +PRDN was more pronounced than improvement in hair count [55].

When compared to placebo, AA-L-PRP and NA-P-PRP sessions resulted in an increase in hair diameter in 2 studies, while no difference in hair diameter was observed when comparing NA-P-PRP to placebo in one study [56,58,59]. Improvement in hair thickness after u-PRP sessions were found significantly milder compared to topical minoxidil [60].

As to patients’ perspective, perceived hair mass was found to be significantly higher in patients treated with NA-P-PRP compared to placebo [59]. Likewise, higher overall patient satisfaction was observed after AA-L-PRP compared to placebo (saline solution) [56]. Improvement in QOL was found to be greater in patients treated with PRP compared to topical minoxidil, in spite of a milder improvement in hair count and thickness [60]. In a patient survey, 58% of patients claimed to be satisfied after PRP treatment, with 65% of them reporting either “marked” or “exceptional” improvement [61].

No serious adverse event was reported after PRP sessions. Mild side effects such as transient edema, itching, tenderness, and transient bleeding at the points of injections were observed in a fraction of subjects.

## 4. Discussion

PRP has been observed to promote remodeling and healing of several tissues, receiving increasing attention by the dermatological community for its potential use in several cutaneous disorders [46]. Since 2012, several studies have been conducted in order to investigate the potential effectiveness of PRP in inducing hair regrowth in a variety of hair diseases [48].

In AGA, the interaction between DHT and androgen receptors on the hair follicles results in the inhibition of the canonical wingless (Wnt)/ß-catenin pathways, resulting in the release of transforming growth factor-B from keratinocytes, apoptosis of the DP cells, and inability to stimulate proliferation of keratinocytes. DHT showed also to inhibit hair growth through blocking the action of growth factors such as insulin-like growth factor 1 (IGF-1) in mouse models. Furthermore, reduced blood flow and oxygen pressure have been observed in AGA, leaving the hair follicle devoid of nutrients [62].

PRP showed to be able to promote proliferation of DP cells in the bulge area and to induce follicular stem cell differentiation into hair follicle cells, stimulating the transition from telogen to anagen phase [49]. The growth factors released after PRP injection are also able to prevent DP cells apoptosis by inducing an increase in Akt and Bcl-2 expression, extending the length of the anagen phase which is typically reduced in AGA. VEGF, PDGF, EGF, TGF-ß, and FGF in PRP showed to be able to foster vascularization, providing oxygen and nutrients to support folliculogenesis [48,63]. Moreover, PRP appears to have a potent anti-inflammatory action, as it promotes the release of mediators such as IL-1 receptor antagonist (IL-1ra), soluble TNF receptors (sTNF-R) I, IL-4, IL-10, IL-13, and interferon γ [64].

Several studies have been conducted investigating the potential effects of PRP in the treatment of AGA in males, with promising results [65,66,67,68,69].

On the contrary, only a few studies investigated the effectiveness of PRP in a female population affected by FAGA.

In a recently published systematic review, Torabi et al. investigated the effect of PRP on FAGA with positive results in terms of hair density, hair diameter, and patients’ satisfaction [70]. However, 4 over 6 included studies were conducted on populations composed by both males and females, with only 2 studies on female-only samples. To our knowledge, this is the first review conducted on populations composed exclusively of female subjects.

In the majority of studies included in our research, an improvement in several hair parameters has been observed. PRP showed superiority over both placebo saline injections and PRDN injections [55,56,58]. This is particularly interesting as scalp injury alone, such as the one performed with saline injections, may induce hair growth by stimulating the release of endogenous growth factors [59].

According to the literature, ideal timing to achieve significant results in both FAGA and MAGA consists of 3 monthly sessions followed by a 3- to 6-month maintenance period [71]. In the study by Puig et al., no significant difference in improvement in hair parameters was detected after a single session of PRP compared to placebo [59]. As suggested by the authors, it is conceivable that one session would not be sufficient to induce a significant clinical effect. This seems to be confirmed by the observations of Tawfik et al. and Dubin et al., who observed superiority of PRP over placebo after a higher number of regularly scheduled sessions [56,58]. These data align with those resulting from studies on MAGA, confirming the proposed timing of sessions.

Interestingly, all included studies observed a significant satisfaction and improvement in quality of life of patients undergoing PRP, which was found greater than placebo and topical minoxidil. Patients were more satisfied if they reported new hair growth as a manifestation of improvement, did not report any adverse reactions, or required 2 or fewer treatments to see improvement [61]. However, satisfaction was also high regardless of clinical results, being found higher in spite of greater efficacy of topical minoxidil in improving hair parameters [60].

As previously suggested, patients’ feelings about hair loss are complex, and perceived efficacy of a given treatment may have a significant impact on their satisfaction and quality of life [60]. The idea of receiving a new, promising treatment such as PRP may positively affect their perception by a placebo-like effect. The autologous nature of PRP may also play a role, being perceived as more “natural” and free of the potential side effects reported for other treatments for FAGA. The relative ease of administration compared to more time-consuming treatments such as topical minoxidil may also result in a greater overall satisfaction. This was observed also in studies conducted on mixed populations of both females and males, such as the randomized, placebo-controlled clinical trial by Shapiro et al. After 3 monthly sessions of PRP, 86% of subjects would recommend the treatment, in spite of the fact that the increase in hair density and hair diameter in the PRP group was not significantly greater than the one observed in the placebo group [65].

Whether PRP effectiveness in the treatment of AGA is influenced by gender is still not elucidated, with conflicting data resulting from studies investigating the effect of PRP in males and/or females. One recent meta-analysis by Gupta et al. suggested milder efficacy in females compared to males, however, this study did not include several reports listed in our review due to insufficient data. Furthermore, many of the analyzed studies were non-randomized, uncontrolled, and had small sample size [72]. Once again, further larger studies reporting males and females as separate populations conducted in a randomized, controlled manner are encouraged to provide stronger evidence on this subject. Additional variability results from the use of different PRP techniques across reports.

The paucity of studies did not allow us to evaluate effectiveness of PRP according to different preparations and techniques. The lack of standardized preparation and technique resulting in high heterogeneity of composition, concentration, dosage, depth, and site of injections make it difficult to compare results.

Overall, the majority of the randomized trials for AGA used activated PRP (A-PRP), with relatively short follow-up. However, it has been hypothesized that non-activated PRP (N-A-PRP) could allow a longer-term response compared to A-PRP. This might be due to saturation of growth factor receptors in the injection sites after A-PRP [68]. Among the studies included in our review, A-PRP was performed in one study. After 28 weeks, a sustained therapeutic response was observed [56]. In the longer study included, sustained significant improvement in hair parameters was observed after 48 weeks from the first session of N-A PRP [60]. Clinical trials comparing long-term efficacy of different PRP protocols in a crossover fashion are required in order to determine the gold-standard of treatment in both male and female AGA and improve clinical outcomes.

We observed significant heterogeneity in assessment methods of hair regrowth, although photography and phototrichograms were the most used. Standardization of quantitative and objective measurements of hair regrowth is needed in order to compare results [57].

In our review, patients enrolled in all the included studies were mostly naïve or underwent a wash-out of a minimum of 2 weeks to a maximum of 1 year. In 2 studies, enrolled patients did not respond to previous treatment with supplements, topical minoxidil, and oral finasteride [57,61]. Only one study compared PRP to topical minoxidil, the current standard of treatment for FAGA. PRP induced only mild improvement in hair thickness compared to minoxidil, however, hair count in the PRP group was found significantly improved, suggesting an enhanced benefit from a combination of the two treatments [60].

Further studies observed a significant improvement in hair parameters in patients treated with both PRP and topical minoxidil or oral finasteride, with the combination of PRP and minoxidil being the most effective in inducing hair regrowth [73,74].

This is thought to result from a synergistic effect of minoxidil and PRP in the increased expression of several growth factors, especially VEGF, resulting in greater angiogenesis and intense neovascularization providing the hair follicle with nutrients and oxygen [63,75].

Patients receiving any topical and/or systemic treatments for AGA are typically excluded from studies on PRP, however, it is worth remarking that in clinical practice most patients considering PRP are already undergoing at least one treatment for their hair loss.

In spite of its promising potential, PRP is not yet included in guidelines for the treatment of FAGA, being performed mostly in a private-practice setting, with considerable financial, emotional, and time investment from the patient. Given their low cost and the favorable safety and efficacy profile, approved drugs for the treatment of FAGA should always be proposed as a first line of therapy in eligible patients. However, due to the growing body of evidence which indicates PRP as a well-tolerated and effective procedure resulting in high levels of satisfaction and quality of life, the authors believe that PRP could be proposed in those patients who did not tolerate or did not respond to these medications. PRP could also represent an additional therapeutic option offered in a combination with topical or oral drugs. Further studies will be needed to determine if platelet concentrates are a valid aid in dermatology and if they can be considered an alternative or support to other therapies [76].

## 5. Conclusions

PRP is a well-tolerated procedure which showed promising results in males-only and mixed populations of AGA patients. Only a few studies have focused on the potential use of PRP in women affected by AGA have been conducted to date.

To date, therapeutic options for women affected by FAGA are scarce. Few studies have been conducted on the effectiveness of PRP for androgenetic alopecia on populations composed only of female subjects, making it difficult to obtain solid information regarding optimal dosage, timing, and administration in these patients.

Evidence from the literature suggests PRP as a potential effective, well-tolerated treatment for FAGA. In spite of milder efficacy compared to topical minoxidil, PRP showed to produce high levels of satisfaction and improvement in the quality of life in patients affected by FAGA. In light of this evidence, PRP could be proposed in patients who did not respond or did not tolerate topical minoxidil, as well as in combination with topical and oral drugs. Further randomized, controlled clinical trials comparing long-term different PRP preparations alone and in combination with approved drugs in a crossover fashion are required in order to develop protocols to improve clinical outcomes in women affected by FAGA. These researches would significantly impact clinical practice, providing patients with new therapeutic options.

## Figures and Tables

**Table 1:** Clinical studies consistent with our research. Study design, number of enrolled patients, Ludwig score, type of intervention, eventual concurrent other treatments, PRP preparation process and technique, number and times of sessions, duration of the study, assessment criteria, endpoints, results, time of follow-up, and reported side effects are described. NR = Not Reported; NA-L-PRP = non-activated leucocyte platelet-rich plasma; NA-P-PRP = non-activated pure platelet-rich plasma; AA-L-PRP = activated leucocyte platelet-rich plasma; NA-PRP = non-activated platelet-rich plasma; u-PRP = not-specified platelet-rich plasma.

Adverse Events	Results	Endpoints		Assessment Methods				Duration of the Study (Weeks)	N° of Sessions	Time Session	PRP Technique		Other Concurrent Treatments		Intervention	Ludwig Stage	Mean Age	N° of Subjects	Type of Study	Year	Author
Transient edema and tenderness (=4), mild itching (N = 2)	Improvement in both hair count and hair diameter in both groups; greater improvement in hair thickness than hair count in Group A compared to Group B.	Hair count; hair diameter		Phototrichograms using Follioscope PT^®^ software at BL and w14				14	1	Single session	60 mL of peripheral blood in g 8 mL of 4% sodium citrate solution centrifuged with the SmartPReP^®^ platelet concentrate system; 4 mL injected in the scalp (intra/perifollicular areas)		No (naïve or 6 months wash-out)		NA-L-PRP followed by 12 weeks of weekly polydeoxirybonucleotide (PDRN) (Group A) vs. 12 weeks of weekly PDRN only (Group B)	NR	33.9 (20–60)	40	Randomized, controlled comparative trial	2015	Lee, S.
NR	No significant difference in the main hair count or hair mass between Group A and B; higher perceived improvement in hair loss in Group A compared to Group B (13.3% vs. 0%); higher perceived hair mass in Group A compared to Group B (26.7% vs. 18.2%)	Hair count; hair mass index; patient satisfaction		Global photography; Cohen hair	check system; patient survey			27	1	Single session	60 mL of peripheral blood centrifuged in Angel PRP system (Cytomedix^®^); produced PRP diluted with platelet-poor plasma; 10 mL of low-concentration, leukocyte- and	erythrocyte-free PRP for injection	No (naïve or 2 months wash-out)		NA-P-PRP (Group A) vs. saline injections (Group B)	II	NR	26	Randomized, double-blind, placebo-controlled trial	2016	Puig, C.
Swollen forehead/face (29%); sore/painful scalp over 3 days (26%)	58% patients claiming to be satisfied with the treatment, with 65% of them reporting either “marked” or “exceptional” improvement.	Patient satisfaction		Custom survey					1 to 3	Every 8–12 weeks	NR		NR; patients who underwent treatment with supplements, topical minoxidil and oral finasteride		u-PRP	NR	51	41	Patient survey	2017	Laird, M.
Temporary pain and pinpoint bleeding at the injection sites	Significant increase in hair density and hair thickness, improvement in hair-pull test in the PRP-treated areas compared with placebo areas; higher overall patient’s satisfaction for PRP areas compared to placebo areas.	Hair density (number of	hairs/cm^2^); hair diameter; hair volume; patient’s satisfaction	Global photography; hair pull test; patient’s satisfaction scale; phototrichograms				28	1 to 4	Every other week	10 mL of peripheral blood in 1.5 mL of sodium citrate solution centrifuged at 1200 rpm for 15 min; buffy coat collected and centrifuged at 2000 rpm for 10 min; lower layer collected		No		AA-L-PRP (Group A) vs. saline injections (Group B)	I-II-III	29.3 (20–45)	30	Randomized, intrapatient split-side, placebo-controlled trial	2018	Tawfik, A.
NR	Significant increase in hair count, hair density and hair diameter; reduction in the vellus hair number	hair count; hair density (frontal-central-vertex); hair diameter; vellus hair count (frontal-central-vertex)		Global photography; hair pull test; phototrichograms using Trichoscan^®^ software				24	4	Every 2 weeks	10 mL of peripheral blood in acid-citrate-dextrose solution in MyCells^®^ system centrifuged at 2500 rpm for 10 min; 5 mL of pure and concentrated autologous platelet-rich plasma injected in the scalp (perifollicular areas)		NR; patients not responding to treatment with	topical minoxidil and/or oral finasteride lasting for at least 1 year	NA-L-PRP	I; I-II; II; II-III	47.1 (33–64)	10	Open-label, not randomized single-group clinical study	2019	Starace, M.
Pain/discomfort/bruising (N = 4)	Greater degree of improvement in hair count in Group B compared to Group A; mild improvement in hair density and cumulative thickness in Group A compared to improvement in Group B. Similar degree of improvement in vellus hair density in both groups; significantly greater improvement in QOL responses in Group A compared to Group B	Hair count; terminal hair density; vellus hair density; total thickness; patient’s quality of life		Phototrichograms using Trichoscan^®^ software; 16-item qualityof	life	(QOL) questionnaire; 7-item questionnaire		48	3	Every 4 weeks; washout among treatment regimens: 8 weeks	60 mL of peripheral blood in 8 mL of citrate dextrose solution centrifuged at 1500 rpm for 10 min followed by 10 further mins at 3500 rpm after removal of the red cell layer; 5 mL of platelet-poor plasma injected in the scalp		No (naïve or 3 months wash-out)		NA-PRP followed by topical minoxidil 1/day for 12 weeks (Group A) vs. topical minoxidil 1/day for 12 weeks then PRP (Group B)	I-II	NR	20	Randomized, controlled, comparative trial	2020	Bruce, AJ
Scalp tightness (N = 10); swelling, redness, post-injection bleeding, tingling (Group A and B)	Significant improvement in hair density in Group A compared to no improvement in Group B; significant improvement in mean hair diameter and global clinical appearance in Group A compared to Group B	Hair density; hair diameter; global clinical appearance		Global photography; magnified photography				24	3	Every 4 weeks	22 mL of peripheral blood in Eclipse^®^ PRP system centrifuged at 3500 rpm for 10 min; removal of the uppermost platelet-poor plasma suprenatant; remaining 4.0 mL platelet-rich plasma injected in the scalp		No (naïve or 12 months wash-out)		NA-L-PRP (Group A) vs. saline injections (Group B)	I-II-III	50 (27–85)	30	Randomized, controlled trial	2020	Dubin, D
/	/	Severity of alopecia; hair count; hair density; hair diameter; anagen to telogen ratio; vellus to terminal ratio; patient’s	quality of life	Severity of Alopecia Tool (SALT) score; phototrichogram using Trichoscan^®^ software; Dermatology Life Quality Index (DLQI)				64 (ensimated)	/	/	/		No (naïve or 2 weeks washout)		/	/	/	16 (enstimated)	Randomized, double blind, intrapatient, split-side, placebo control trial	2021 (enstimated)	ClinicalTrials.gov identifier: NCT03474718

## References

[1] Jahoda C.A. (1998). Cellular and developmental aspects of androgenetic alopecia. Exp. Dermatol..

[2] Olsen E.A. (2001). Female pattern hair loss. J. Am. Acad. Dermatol..

[3] Norwood O.T., Lehr B. (2000). Female Androgenetic Alopecia: A Separate Entity. Dermatol. Surg..

[4] Price V.H. (2003). Androgenetic alopecia in women. J. Investig. Dermatol. Symp. Proc..

[5] Piraccini B.M., Alessandrini A. (2014). Androgenetic alopecia. G Ital. Dermatol. E Venereol..

[6] Shannon F., Christa S., Lewei D., Carolyn G. (2015). Demographics of women with female pattern hair loss and the effectiveness of spironolactone therapy. J. Am. Acad. Dermatol..

[7] Ellis J.A., Stebbing M., Harrap S.B. (2001). Polymorphism of the androgen receptor gene is associated with male pattern baldness. J. Investig. Dermatol..

[8] Tu Y.-A., Lin S.-J., Chen P.-L., Chou C.-H., Huang C.-C., Ho H.-N., Chen M.-J. (2019). HSD3B1 gene polymorphism and female pattern hair loss in women with polycystic ovary syndrome. J. Formos Med. Assoc..

[9] Redler S., Birch M., Drichel D., Dobson K., Brockschmidt F., Tazi-Ahnini R., Giehl K., Kluck N., Kruse R., Lutz G. (2011). Investigation of variants of the aromatase gene (CYP19A1) in female pattern hair loss. Br. J. Dermatol..

[10] Redler S., Messenger A.G., Betz R.C. (2017). Genetics and other factors in the aetiology of female pattern hair loss. Exp. Dermatol..

[11] Seleit I., Bakry O.A., Badr E., Mabrouk M. (2020). Vitamin D Receptor Gene Polymorphisms Taq-1 and Cdx-1 in Female Pattern Hair Loss. Indian J. Dermatol..

[12] Yip L., Rufaut N., Sinclair R. (2011). Role of genetics and sex steroid hormones in male androgenetic alopecia and female pattern hair loss, an update of what we now know. Australas J. Dermatol..

[13] Randall V.A. (2008). Androgens and hair growth. Dermatol. Ther..

[14] Itami S., Inui S. (2005). Role of androgen in mesenchymal epithelial interactions in human hair follicle. J. Investig. Dermatol. Symp. Proc..

[15] Kwack M.H., Sung Y.K., Chung E.J., Im S.U., Ahn J.S., Kim M.K., Kim J.C. (2008). Dihydrotestosterone-inducible dickkopf 1 from balding dermal papilla cells causes apoptosis in follicular keratinocytes. J. Investig. Dermatol..

[16] Winiarska A., Mandt N., Kamp H., Hossini A., Seltmann H., Zouboulis C., Blume-Peytavi U. (2006). Effect of 5alpha-dihydrotestosterone and testosterone on apoptosis in human dermal papilla cells. Skin Pharmacol. Physiol..

[17] Norwood O.T. (2001). Incidence of female androgenetic alopecia (female pattern alopecia). Dermatol. Surg..

[18] Vexiau P., Chaspoux C., Boudou P., Fiet J., Abramovici Y., Rueda M., Hardy N., Reygagne P. (2000). Role of Androgens in Female-Pattern Androgenetic Alopecia, Either Alone or Associated With Other Symptoms of Hyperandrogenism. Arch. Dermatol. Res..

[19] Olsen E.A. (2005). Female Pattern Hair Loss and its Relationship to Permanent/Cicatricial Alopecia: A New Perspective. J. Investig. Dermatol. Symp. Proc..

[20] El-Husseiny R.M., Saleh H.M., Moustafa A.A., Salem S.A. (2020). Comparison between single—Versus double-spin prepared platelet-rich plasma injection in treatment of female pattern hair loss: Clinical effect and relation to vascular endothelial growth factor. Arch. Dermatol. Res..

[21] Sawaya M.E., Price V.H. (1997). Different levels of 5alpha-reductase type I and II, aromatase, and androgen receptor in hair follicles of women and men with androgenetic alopecia. J. Investig. Dermatol..

[22] Ludwig E. (1977). Classification of the types of androgenetic alopecia (common baldness) occurring in the female sex. Br. J. Dermatol..

[23] Rathnayake D., Sinclair R. (2010). Innovative use of spironolactone as an antiandrogen in the treatment of female pattern hair loss. Dermatol. Clin..

[24] Schmidt J.B., Lindmaier A., Trenz A., Schurz B., Spona J. (1991). Hormone studies in females with androgenic hairloss. Gynecol. Obstet. Investig..

[25] Orme S., Cullen D.R., Messenger A.G. (1999). Diffuse female hair loss: Are androgens necessary?. Br. J. Dermatol..

[26] Starace M., Orlando G., Alessandrini A., Piraccini B.M. (2020). Female Androgenetic Alopecia: An Update on Diagnosis and Management. Am. J. Clin. Dermatol..

[27] Russo P.M., Fino E., Mancini C., Mazzetti M., Starace M., Piraccini B.M. (2019). HrQoL in hair loss-affected patients with alopecia areata, androgenetic alopecia and telogen effluvium: The role of personality traits and psychosocial anxiety. J. Eur. Acad. Dermatol. Venereol. JEADV.

[28] Sinclair R., Jolley D., Mallari R., Magee J. (2004). The reliability of horizontally sectioned scalp biopsies in the diagnosis of chronic diffuse telogen hair loss in women. J. Am. Acad. Dermatol..

[29] Ross E.K., Vincenzi C., Tosti A. (2006). Videodermoscopy in the evaluation of hair and scalp disorders. J. Am. Acad. Dermatol..

[30] Rakowska A., Slowinska M., Kowalska-Oledzka E., Olszewska M., Rudnicka L. (2009). Dermoscopy in female androgenic alopecia: Method standardization and diagnostic criteria. Int. J. Trichol..

[31] Mubki T., Rudnicka L., Olszewska M., Shapiro J. (2014). Evaluation and diagnosis of the hair loss patient: Part II. Trichoscopic and laboratory evaluations. J. Am. Acad. Dermatol..

[32] Ummiti A., Priya P.S., Chandravathi P.L., Kumar C.S. (2019). Correlation of Trichoscopic Findings in Androgenetic Alopecia and the Disease Severity. Int. J. Trichol..

[33] Kanti V., Messenger A., Dobos G., Reygagne P., Finner A., Blumeyer A., Trakatelli M., Tosti A., Del Marmol V., Piraccini B. (2018). Evidence-based (S3) guideline for the treatment of androgenetic alopecia in women and in men—Short version. J. Eur. Acad. Dermatol. Venereol. JEADV.

[34] Van Neste D. (2006). Female patients complaining about hair loss: Documentation of defective scalp hair dynamics with contrast-enhanced phototrichogram. Skin Res. Technol..

[35] Rushton D.H., de Brouwer B., de Coster W., van Neste D.J. (1993). Comparative evaluation of scalp hair by phototrichogram and unit area trichogram analysis within the same subjects. Acta Derm. Venereol..

[36] Van Neste D.J. (2001). Contrast enhanced phototrichogram (CE-PTG): An improved non-invasive technique for measurement of scalp hair dynamics in androgenetic alopecia--validation study with histology after transverse sectioning of scalp biopsies. Eur. J. Dermatol. EJD.

[37] Hoffmann R. (2001). TrichoScan: Combining epiluminescence microscopy with digital image analysis for the measurement of hair growth in vivo. Eur. J. Dermatol. EJD.

[38] Riedel-Baima B., Riedel A. (2009). Use of the TrichoScan to assess female pattern hair loss. Dermatol. Surg..

[39] López V., Martín J.M., Sánchez R., Ortega C., Ricart J.M. (2011). Usefulness of TrichoScan professional in the evaluation of hair loss in females. Report of 180 cases. J. Eur. Acad. Dermatol. Venereol. JEADV.

[40] Gassmueller J., Rowold E., Frase T., Hughes-Formella B. (2009). Validation of TrichoScan technology as a fully-automated tool for evaluation of hair growth parameters. Eur. J. Dermatol. EJD.

[41] Carmina E., Azziz R., Bergfeld W., Escobar-Morreale H.F., Futterweit W., Huddleston H., Lobo R., Olsen E. (2019). Female Pattern Hair Loss and Androgen Excess: A Report From the Multidisciplinary Androgen Excess and PCOS Committee. J. Clin. Endocrinol. Metab..

[42] Suchonwanit P., Thammarucha S., Leerunyakul K. (2019). Minoxidil and its use in hair disorders: A review. Drug Des. Dev. Ther..

[43] Gentile P., Dionisi L., Pizzicannella J., De Angelis B., De Fazio D., Garcovich S. (2020). A randomized blinded retrospective study: The combined use of micro-needling technique, low-level laser therapy and autologous non-activated platelet-rich plasma improves hair re-growth in patients with androgenic alopecia. Expert Opin. Biol. Ther..

[44] Hesseler M.J., Shyam N. (2020). Platelet-Rich Plasma and Its Utilities in Alopecia: A Systematic Review. Dermatol. Surg..

[45] Ehrenfest D.M.D., Bielecki T., Mishra A., Borzini P., Inchingolo F., Sammartino G., Rasmusson L., Evert P.A. (2012). In Search of a Consensus Terminology in the Field of Platelet Concentrates for Surgical Use: Platelet-Rich Plasma (PRP), Platelet-Rich Fibrin (PRF), Fibrin Gel Polymerization and Leukocytes. Curr. Pharm. Biotechnol..

[46] Mercuri S.R., Vollono L., Paolino G. (2020). The Usefulness of Platelet-Rich Plasma (PRP) for the Treatment of Vitiligo: State of the Art and Review. Drug Des. Dev. Ther..

[47] Dhurat R., Sukesh M. (2014). Principles and Methods of Preparation of Platelet-Rich Plasma: A Review and Author’s Perspective. J. Cutan Aesthetic Surg..

[48] Gupta A.K., Carviel J.A. (2016). Mechanistic Model of Platelet-Rich Plasma Treatment for Androgenetic Alopecia. Dermatol. Surg..

[49] Li Z.J., Choi H.-I., Choi D.-K., Sohn K.-C., Im M., Seo Y.-J., Lee Y.-H., Lee J.-H. (2012). Autologous Platelet-Rich Plasma: A Potential Therapeutic Tool for Promoting Hair Growth. Dermatol. Surg..

[50] Pierce G.F., A Mustoe T., Lingelbach J., Masakowski V.R., Griffin G.L., Senior R.M., Deuel T.F. (1989). Platelet-derived growth factor and transforming growth factor-beta enhance tissue repair activities by unique mechanisms. J. Cell Biol..

[51] Mecklenburg L., Tobin D.J., Müller-Röver S., Handjiski B., Wendt G., Peters E.M., Pohl S., Moll I., Paus R. (2000). Active Hair Growth (Anagen) is Associated with Angiogenesis. J. Investig. Dermatol..

[52] Giudice A., Esposito M., Bennardo F., Brancaccio Y., Buti J., Fortunato L. (2019). Dental extractions for patients on oral antiplatelet: A within-person randomised controlled trial comparing haemostatic plugs, advanced-platelet-rich fibrin (A-PRF+) plugs, leukocyte- and platelet-rich fibrin (L-PRF) plugs and suturing alone. Int. J. Oral Implant.

[53] Moher D., Liberati A., Tetzlaff J., Altman D.G., PRISMA Group (2009). Preferredreporting items for systematic reviews and meta-analyses: The PRISMAstatement. BMJ.

[54] Gupta M., Mysore V. (2016). Classifications of Patterned Hair Loss: A Review. J. Cutan Aesthetic Surg..

[55] Lee S.H., Zheng Z., Kang J.S., Kim D.Y., Oh S.H., Cho S.B. (2015). Therapeutic efficacy of autologous platelet-rich plasma and polydeoxyribonucleotide on female pattern hair loss. Wound Repair Regen..

[56] Tawfik A.A., Osman M.A.R. (2018). The effect of autologous activated platelet-rich plasma injection on female pattern hair loss: A randomized placebo-controlled study. J. Cosmet. Dermatol..

[57] Starace M., Alessandrini A., D’Acunto C., Melandri D., Bruni F., Pr A.P., Pr B.M.P. (2018). Platelet-rich plasma on female androgenetic alopecia: Tested on 10 patients. J. Cosmet. Dermatol..

[58] Dubin D.P., Lin M.J., Leight H.M., Farberg A.S., Torbeck R.L., Burton W.B., Khorasani H. (2020). The effect of platelet-rich plasma on female androgenetic alopecia: A randomized controlled trial. J. Am. Acad. Dermatol..

[59] Puig C.J., Reese R., Peters M. (2016). Double-Blind, Placebo-Controlled Pilot Study on the Use of Platelet-Rich Plasma in Women with Female Androgenetic Alopecia. Dermatol. Surg..

[60] BBruce A.J., Pincelli T.P., Heckman M.G., Desmond C.M., Arthurs J.R., Diehl N.N., Douglass E.J., Bruce C.J., Shapiro S.A. (2020). A Randomized, Controlled Pilot Trial Comparing Platelet-Rich Plasma to Topical Minoxidil Foam for Treatment of Androgenic Alopecia in Women. Dermatol. Surg..

[61] Laird M.E., Sicco K.I.L., Reed M.L., Brinster N.K. (2018). Platelet-Rich Plasma for the Treatment of Female Pattern Hair Loss: A Patient Survey. Dermatol. Surg..

[62] Goldman B.E., Fisher D.M., Ringler S.L. (1996). Transcutaneous Po2 of the Scalp in Male Pattern Baldness: A New Piece to the Puzzle. Plast. Reconstr. Surg..

[63] Li W., Enomoto M., Ukegawa M., Hirai T., Sotome S., Wakabayashi Y., Shinomiya K., Okawa A. (2012). Subcutaneous Injections of Platelet-Rich Plasma into Skin Flaps Modulate Proangiogenic Gene Expression and Improve Survival Rates. Plast. Reconstr. Surg..

[64] Gkini M.-A., Kouskoukis A.-E., Tripsianis G., Rigopoulos D., Kouskoukis K. (2014). Study of platelet-rich plasma injections in the treatment of androgenetic alopecia through an one-year period. J. Cutan. Aesthetic Surg..

[65] Butt G., Hussain I., Ahmed F.J., Choudhery M.S. (2019). Efficacy of platelet-rich plasma in androgenetic alopecia patients. J. Cosmet Dermatol..

[66] Qu Q., Shi P., Yi Y., Fan Z., Liu X., Zhu D., Chen J., Ye K., Miao Y., Hu Z. (2019). Efficacy of Platelet-rich Plasma for Treating Androgenic Alopecia of Varying Grades. Clin. Drug Investig..

[67] Schiavone G., Raskovic D., Greco J., Abeni D. (2014). Platelet-Rich Plasma for Androgenetic Alopecia. Dermatol. Surg..

[68] Shapiro J., Ho A., Sukhdeo K., Yin L., Sicco K.L. (2020). Evaluation of platelet-rich plasma as a treatment for androgenetic alopecia: A randomized controlled trial. J. Am. Acad. Dermatol..

[69] Alves R., Grimalt R. (2016). Randomized Placebo-Controlled, Double-Blind, Half-Head Study to Assess the Efficacy of Platelet-Rich Plasma on the Treatment of Androgenetic Alopecia. Dermatol. Surg..

[70] Torabi P., Behrangi E., Goodarzi A., Rohaninasab M. (2020). A systematic review of the effect of platelet-rich plasma on androgenetic alopecia of women. Dermatol. Ther..

[71] Gupta A.K., Cole J., Deutsch D.P., Everts P.A., Niedbalski R.P., Panchaprateep R., Rinaldi F., Rose P.T., Sinclair R., Vogel J.E. (2019). Platelet-Rich Plasma as a Treatment for Androgenetic Alopecia. Dermatol. Surg..

[72] Gupta A.K., Renaud H.J., Bamimore M. (2020). Platelet-rich plasma for androgenetic alopecia: Efficacy differences between men and women. Dermatol. Ther..

[73] Ho A., Sukhdeo K., Lo Sicco K., Shapiro J. (2020). Trichologic response of platelet-rich plasma in androgenetic alopecia is maintained during combination therapy. J. Am. Acad. Dermatol..

[74] Alves R., Grimalt R. (2018). Platelet-Rich Plasma in Combination With 5% Minoxidil Topical Solution and 1 mg Oral Finasteride for the Treatment of Androgenetic Alopecia: A Randomized Placebo-Controlled, Double-Blind, Half-Head Study. Dermatol. Surg..

[75] Lachgar S., Charveron M., Gall Y., Bonafe J.L. (1998). Minoxidil upregulates the expression of vascular endothelial growth factor in human hair dermal papilla cells. Br. J. Dermatol..

[76] Bennardo F., Bennardo L., Del Duca E., Patruno C., Fortunato L., Giudice A., Nisticò S.P. (2020). Autologous platelet-rich fibrin injections in the management of facial cutaneous sinus tracts secondary to medication-related osteonecrosis of the jaw. Dermatol. Ther..

